# A qualitative analysis of public health officials' experience in California during COVID-19: priorities and recommendations

**DOI:** 10.3389/fpubh.2023.1175661

**Published:** 2023-09-13

**Authors:** Rita V. Burke, Anna S. Distler, Timothy C. McCall, Emma Hunter, Shruti Dhapodkar, Larissa Chiari-Keith, Aaron A. Alford

**Affiliations:** ^1^Population and Public Health Sciences, Keck School of Medicine, University of Southern California, Los Angeles, CA, United States; ^2^National Association of County and City Health Officials, Washington, DC, United States; ^3^Department of Clinical Research and Leadership, The George Washington University, Washington, DC, United States; ^4^San Mateo County Health, San Mateo, CA, United States; ^5^Alala Advisors, LLC., Irvine, CA, United States

**Keywords:** COVID-19, harassment, violence, public health, California, doxing

## Abstract

**Objectives:**

The aim of this study was to collect qualitative data regarding the violence faced by public health officials during the COVID-19 pandemic and create a guideline of recommendations to protect this population moving forward.

**Methods:**

Two focus groups were conducted virtually from April 2022 to May 2022. All nine participants were public health officials from across California. A grounded theory approach was used to analyze the data from these focus groups.

**Results:**

The main recurrent experiences among public health officials were harassment, psychological impact, systemic backlash, and burnout. Several recommendations for supporting public health officials were highlighted, including security and protection, mental health support, public awareness, and political/institutional support.

**Conclusion:**

Our study captures the violent experiences that health officials have faced during the COVID-19 pandemic. To maintain the integrity of the public health system, timely changes must be made to support and protect health officials. Our guideline of recommendations provides a multi-faceted approach to the urgent threats that officials continue to face. By implementing these solutions, we can strengthen our public health system and improve our response to future national emergencies.

## Introduction

Throughout the past 3 years, the COVID-19 pandemic has sparked several unforeseen circumstances, such as violence, stigma, and the politicization of the public health system in the United States (1, 2). The backbone of this system is comprised of public health personnel who have worked tirelessly to prevent the spread of COVID-19 (3). Prior to the pandemic, the public health system was weakened by systematic flaws and chronic underfunding (4). Between 2003 and 2021, funding for Public Health Emergency Preparedness dropped by approximately 50% after adjusting for inflation (5). Along with being fiscally unsupported, the pandemic has exposed several weaknesses across all facets of the healthcare and public health system (6). Between 2020 and 2021, USA Facts reported a total of 101,918 COVID-19-related deaths in California and an estimated 11,263,545 reported cases. These staggering numbers further strained healthcare workers, including public health officials. Furthermore, healthcare personnel have faced tremendous violence due to the political frustration of citizens nationally, who feel that their personal liberties have been encroached (3, 6–8). Dye et al. found that, even after controlling for various confounders, healthcare workers are significantly more likely to experience COVID-19-related bullying or stigma compared to others (1).

Public health officials comprise a particularly vulnerable subset of this population and have faced extreme violence during the COVID-19 pandemic (6, 7, 9). Two survey waves reported that American adults who believed that harassment of health officials due to business closures was justified rose from 20% to 25% in November 2020 and from 15% to 21% from July 2021 to August 2021 (10). Harassment includes death threats, vandalism, hate speech, and physical endangerment (9). In 2020, an estimated 9%−12% of public health officials reported receiving either individual or family threats (11).

There have been numerous breaches of officials' personal information as their residential addresses, phone numbers, and emails were doxed through the Internet (12, 13). Many officials fear the loss of their jobs or putting themselves at further risk, leaving them silent and isolated (7, 9, 14). This also pressures officials to comply with public or political opinions rather than focusing on what is best for community health (7). Officials who are harassed are vulnerable to stress intolerance, a lack of meaning in work, and greater turnover rates (15, 16). These conditions also contribute to mental illness among officials during and following the pandemic's heights (16).

Bryant et al. reported that 53% of public health workers had reported feeling at least one symptom of mental illness in the previous 2 weeks (14). Anxiety, depression, and post-traumatic stress disorder (PTSD) were among the most prevalent conditions (14). Despite suffering from these illnesses, officials continued working 12–14 h per day with a minimal number of employees and negligible support (17).

Attrition rates among public health officials have increased since the first spike of retirements in May 2020 (18). Ward et al. reported that from March 2020 to January 2021, a total of 120 resignations, 58 retirements, 20 firings, and 24 other departures occurred among health officials in the United States (18). Some public health experts claim that this is the greatest exodus of public health workers throughout American history, with 1 in 6 Americans losing a local public health official during the pandemic (19).

The public health system will further suffer, along with its officials, if timely changes are not made to prevent violence moving forward (13, 20). Topazian et al. highlighted strategies to support health officials, such as additional funding, more protection, and an improved system against violence (10, 16). Furthermore, Ward et al. reported that, in order to understand violence among public health officials, affected individuals must have a platform to share their personal experiences (18).

The 2020 Forces of Change Survey Report by the National Association of County and City Health Officials, along with other literature, captures quantitative data showing the violence toward public health officials during the pandemic (11). However, there is a lack of qualitative research on this topic. The goal of our study was to fill this research gap and provide public health officials with the opportunity to openly discuss their experiences during the pandemic. In August 2022, the *American Journal of Public Health* produced a podcast that shed light on the experiences of public health officials during the pandemic (12). Our study expands on several of the issues that were discussed, such as the negative and abrupt spotlight on public health, the isolation of officials, and the politicization of the pandemic. Furthermore, through collaboration with public health personnel, we will create a robust guideline of changes and recommendations to better support and protect this targeted population in the future. We hope that these proposed solutions will also shape political and institutional actions moving forward.

## Methods

This qualitative study was a collaborative effort between the University of Southern California, a local health officers' organization, and the National Association of City and County Health Officials. Institutional Review Board approval was received to collect quantitative and qualitative data from public health officials from across California. A total of two focus groups were held, with a total of nine participants overall. These were conducted between April 2022 and May 2022.

### Population

A total of nine English-speaking public health officials took part in this study. Participants were recruited through an organization of health officers in California via electronic approaches such as email. No participants dropped out of the study.

### Measures

Before the focus groups, participants answered a short demographic survey that assessed officials' duration of public health experience and their contemplations on holding their positions during the pandemic. Focus groups were then led by a senior researcher (R.V.B.), who facilitated the discussion with the officials. A research assistant (A.S.D.) was also present in the focus groups to take notes. The researchers had no prior connections with the participants before the focus groups were conducted. Participants were informed that the goal of the project was to understand the experiences of public health officials during the pandemic and to propose solutions to any negative experiences. An outline of the interviewers' script for each focus group is provided in the Appendix and includes the introduction, questions, and closing statement.

Two focus groups lasting ~1 h and a half each were conducted, with three to six participants in each. Each participant was present for only one of these groups. Focus groups were kept small to ensure that participants could speak freely in private settings. Additional focus groups were not conducted since this was a pilot study, and more subjects are anticipated to be added in the future. Our sample size was limited because the officials were overwhelmed and stressed and often struggling with mental illnesses such as anxiety, depression, and PTSD. Both focus groups were conducted via Zoom using audio and video recording, with informed consent from all participants.

### Statistical analysis

A grounded theory approach, established by Glaser et al. (21), was used to analyze the qualitative data. This strategy was utilized because it is a flexible approach that can cope with highly exploratory scenarios where little is known. To analyze the data, the discussion from each focus group was transcribed, and participants were deidentified. These transcripts were kept confidential and viewed only by the primary coder (A.S.D.) and the principal investigator (R.V.B.). The data from the demographic surveys were organized using Microsoft Excel. The primary coder (A.S.D.) established codes to summarize the data on ATLAS.ti (Version 9.1.3; ATLAS.ti Scientific Software Development GmbH, Berlin, Germany). A conventional content analysis approach was used to organize the rich qualitative data from the focus groups. To ensure that the coding was both valid and reliable, the criteria for assigning a specific code to a block of text were systematically developed and well documented using the ATLAS.ti software. The coding scheme was refined and expanded upon to reflect and include emerging insights throughout the coding process. Twenty percent of the coder's cases were double-coded by another coder to ensure continued inter-coder reliability and validity. Disparities in coding were discussed and resolved. These codes lead to the development of larger concepts. There was a simultaneous and iterative process of data collection, analysis, and theory construction that resulted in adaptive changes in the focus groups as the study progressed. In Tables 2, 3, various quotes were mentioned in bold to highlight their importance and significance within each concept.

## Results

The participants' demographic information is summarized in Table 1. All the participants had 5 or more years of experience working in the public health field, with a majority (66%) having 10+ years of experience. Over half (56%) of the participants had been in their most recent job for 1–3 years, while the remainder had worked at their jobs for more than 4 years. Seven (78%) participants had considered leaving their most recent position. Six (67%) participants reported experiencing some form of harassment in their most recent position, compared to three (33%) participants who did not.

**Table 1:** Demographic characteristics of focus group participants.

**Average age (years)**	**54.2 (SD 8.33)**	
Gender	2 males	7 females	
		N	Percentage (%)
Ethnicity	Black	0	0
	Hispanic	2	22
	White	4	44
	Asian	3	33
	Other	0	0
Years in most recent job	1–3	5	56
	4–7	1	11
	8–10	3	33
Years in the field	0–5	0	0
	6–10	3	33
	11–16	2	22
	17–21	2	22
	22–40	2	22
Children	Yes	6	67
	No	3	33
Have you experienced ANY harassment in your current/most recent position?	Yes	6	67
	No	3	33
Have you thought about leaving your current/most recent position?	Yes	7	78
	No	2	22

The qualitative data displayed several repetitive themes among the experiences of public health officials. These themes included (1) harassment, (2) psychological impact, (3) systemic backlash, and (4) burnout.

### Experiences among public health officials

#### Harassment

Within the past 3 years, the general public has associated health officials with the negative impact of the COVID-19 pandemic. In response, there were several acts of violence toward officials. Furthermore, angry protestors have knocked down officials' doors, invaded their personal privacy, and committed other forms of threatening harassment. Evidence shows that female public health officials have been treated with disproportionally more violence than their male counterparts. Although officials' assistants are trained to screen text messages and emails for threatening messages, officials often still receive these threats (Table 2, Theme 4).

**Table 2:** Recurrent experiences of public health officers.

**Theme (subtheme)**	**Quotes**
**Harassment**	
1. Hate speech	“…people get to stand outside your house and yell to your children, “Your parent is a murderer”. It's free speech. And they just sit by. Every week, “Murderer, murderer, murderer, your parent's a murderer”. That shouldn't be okay. And it shouldn't be okay at a board meeting to take up the public's time for at a time with just, it's not topical. It's just venting, though.”
	“I think about poor, redirected state workers who never had anything to do with public health and had to take just all of the abuse from every person that they called. All these people who, they went into public health because they wanted to do like dental hygiene. Here they are getting harassed in the grocery store…”
	“I mean, there are emails where people just basically say I don't know what I'm doing and that I'm an idiot and list all the things that I have done wrong…”
	“I think this is kind of part of a national conversation about when is it free speech and when is it hate speech? I've received plenty of comments at board meetings and committee meetings and emails that feel hate speech to me, that feels like, that's not right. But it just is like, well, this is free speech. People get to do that.”
2. Gender discrimination	From a male official, about a female official: “I took an extended leave where my deputy health officer took over during a period where we'd just put in a mandatory vaccination for our first responders. It was kind of a case-control situation in some ways, because the way people spoke to her was different than the way they spoke to me…She was never frankly threatened in the way that others have been, but it just seemed like the permission people gave themselves to just be vitriolic in public ways was different.”
	“Yeah, I told them, within 6 weeks, that I wouldn't extend my time with them, but that I would try to stay on until they found a replacement, but it just got more and more vile. Super misogynistic, very threatening to all of the women in the department and me, as a woman who's a health officer. Then, there was a male health officer in the county to the south of us who got none of that.”
	“…a lot of it was just getting back to gender. If you're sort of looking for a deeper dive into that particular dynamic, it is kind of an interesting situation where I had a woman for a bit and a man for a bit, and it really did differ.”
3. Invasion of personal privacy	“I will never be a health officer because I will never, ever want to be in a position where I can be threatened and harassed in that way, and I very much look forward to just being a cog in the wheel for the rest of my career.”
	“That's when I got really scared because an alarm system had been put in my home, and even my kids were freaking out because I was getting all these calls on my personal cellphone.”
	“…then protestors started coming to our homes, and they kind of pushed the door in against my daughter at one point when she was home alone.”
**Harassment**	
4. Internet threats	“Subsequently, it definitely got worse, and there was a lot of really negative emails, threats. There were a few death threats.”
	“This person who emails regularly, basically was saying to me and the board that ‘You all should apologize for all of the harm that you've created over the past two years.”'
	“I would get harassing emails and text messages at all hours of the day and night from the Board of Supervisors, who were not supportive and from the community in general. Lots of members of the community would make all sorts of threats all over Facebook to the department. It pretty quickly got to the point where I felt unsafe doing anything other than going to the office and being at home.”
**Psychological impact**	
5. Fear	“I think we're privileged [here] that most of us have a supportive board of supervisors, CIOs, or CEOs, whatever you have in your county, and supportive sheriffs. But much of the rest of the state, that is not the case. There's a lot of animosity between the health officers, so they can't talk. They can't share. They're afraid to share their experiences.”
	“…We went to a restaurant, and I was confronted by one of the anti-vaxxers, he was the reason why I stopped going to board meetings because he pretty much accosted me in the parking lot…He's a very tall, big, muscular guy, and so I had stopped showing up to board meetings in person because I felt very threatened.”
6. Trauma (PTSD)	“…And so, it was all part of the same group's plans to draw attention to their platform, but that made us feel we really needed to protect ourselves. And it made us more worried for our kids and our families because our kids were called out by name, both my director and I.”
	“I'm super protective of my privacy and just want to be a totally anonymous person…I would not want to be the public face ever of anything. Even if I come up with some brilliant chronic disease program, I'll be like, “Somebody else gets to be the face of this program,” because it's not worth it to me to feel physically unsafe that way.”
**Systemic backlash**	
7. Masking and vaccine mandates	“As people mentioned here, we had to build up these massive vaccine administration mechanisms, and it took a ton of work for everyone, and everybody was complaining about they couldn't get their things fast enough. We had all of these weaknesses that put us in a super weak position to begin with, and then we're blamed for it as if we could have done anything in the super broken system.”
	“I also think that it does ebb and flow with the policies, like when we still had a mask mandate in place where surrounding counties didn't, there was sort of a fever pitch of loud, angry folks… Then there's just a baseline level of crazy.”
8. Lack of political enforcement	“To come into this local politics, which is just so polarized and negative, especially at this time, was incredible.”
	“Pretty quickly, I would say within the first 6 weeks, I knew that there was no chance that anyone was going to support us, that the County Counsel would give the correct, textbook legal advice of, “Yes, you have this authority', but would not have been willing to go to court over it.”
**Burnout**	
9. High attrition rates	“Also, the PRA requests have been like we've never seen, and it takes up a lot of staff time to answer all that. It's a big time suck, and so I think it's just the ongoing workload, and attrition of staff. I've been without a director of public health nursing for a year and a half, so it's just the churn of just traumatized staff who decide to retire, and then you just can't hire people, so that part has just been in addition to everything else.”
	“I went to residency with several other people who also went into public health in California and in other states, and every one of us who was in a public-facing position has left that public-facing position during the course of this pandemic because we've all experienced threats, and some of them have left public health entirely because of that.”
	“…it's getting incredibly harder and harder to let these things just roll off…the extent of the craziness, I never thought. Frankly, yeah, I'm definitely looking for other jobs…”
	“…but what we're dealing with now is just how are you going to hire people? I mean, I think people are burned out. You might see retirements, and sabbaticals, and people just decide they're going to change their career trajectory.”
	“Even community health workers, and everybody. Everywhere, there's attrition right now. That's why I said, I think local public health is in big, big trouble for just this.”
10. Exhaustion as a public figure	“I love public health, but I hate being a public figure…I find it to be soul-sucking. I didn't go into this to be somebody that's going to be recognized in the back of my head, and yeah, I didn't sign up for this part. I like the work, but I don't want this role...”
11. Lack of encouragement	“I think that public health has always been kind of like in the background, just doing all of this stuff nobody really knows about. 'Your water is safe. We're testing it. Your milk. We're dealing with these outbreaks'. These things, nobody knows about. I think what this pandemic has done is, potentially, brought some of the negative things about what public health is responsible for to light, to a point where I don't know how we're going to fix it.”
	“Yeah, we're supposed to be apolitical. We're supposed to be looking at the data and making data-driven decisions, and I tried, and have still tried to the best of my ability, to do the data-driven approach, and back it up, and stick to it regardless of what comes…I've done it now for this long, and I don't how much longer I can do this.”

#### Psychological impact

Public health officials suffered from many mental illnesses such as PTSD, anxiety, and depression. Harassment from citizens instilled fear in officials for their personal safety and their families' safety. Respondents reported that this fear kept them isolated from their communities and neighborhoods. Many officials reported losing friends during the pandemic due to their grueling work schedules and the isolation of holding public health positions. Lastly, some officials reported that they could not sit still as their anxiety was too overpowering. They stated that these conditions were not sustainable and contributed to high levels of burnout.

#### Systemic backlash

The systemic backlash from Administrative Board Members and government institutions made it difficult for officials to do their jobs properly. Particularly, there was often backlash due to masking and vaccine mandates, and public health officials were faced with the brunt of this hostility. Officials were often unsupported by political leaders and law enforcement, which restricted their autonomy and power (Table 2, Themes 6 and 7).

#### Burnout

Attrition rates have heightened as many experienced individuals were exhausted and overworked. Officials stepped down from public-facing positions that were constantly targeted by the public. Many who had not yet quit were searching for other job opportunities since the beginning of the pandemic. After 10+ years working in public health, many officials were pessimistic about the future of this field.

### Recommendations and solutions

Throughout the focus groups, officials spoke about several recommendations that could better support and protect them in the future.

#### Security and protection

Officials believed that improved security would make them feel safer and improve mental illness. First, law enforcement authorities must make a greater effort to protect officials from the doxing of their personal information. This can be accomplished by sweeping the Internet of personal emails, phone numbers, and addresses to mitigate threats that officials receive. Health departments should be equipped with resources to implement security protections or hire personnel who have the skills to do so. Lastly, protection of personal residential addresses would prevent angry protestors from storming officials' houses and properties. Quote 3 in Theme 2 shows how aggression and hostility can also transcend the workplace, illustrating the need for protection in other environments as well (Table 3).

**Table 3:** Recommendations and solutions.

**Theme (subtheme)**	**Quotes**
**Security and protection**	
1.Personal information	“What would be really helpful is to really have some assistance with getting everything personal off internet, off of social media sites, off of whatever it is, because I've had to pay for services to try and just get myself off of Spokeo and PeopleFinders…That's why people have my personal cellphone, because they're able to get it somewhere. If there's some way to do something to help us get our stuff off any general networks where people can find information on us, that would be great.”
	“I don't know if it's even possible, but I know the governor did endorse some bill or other to keep our personal information, and location, and things like that safe. It doesn't work. It's not worked.”
	“I've had an escort when I, whenever I want or feel like I need an escort from the Sheriff's Office, they've been very supportive, but the other things that we've done, which all seem Band-Aids are that my administrative assistant was trained to screen my emails for any kind of email that might be traumatizing to me.”
2. Physical safety	“I still have a share of patrols on my house, four times a day on the front and the back. I'm on a locked street, but they've broken down the gate a number of times.”
	“It quickly got to the point where I felt unsafe doing anything other than going to the office and being at home. The only reason I wasn't harassed at my residence was because I didn't own the property. There was no public record of me being there because I had come back temporarily.”
	“The thing that made me more paranoid is I was out at a restaurant for my colleague's baby shower, and I helped to organize it. We went to a restaurant and I was confronted by one of the anti-vaxxers...He was the reason why I stopped going to board meetings because he pretty much accosted me in the parking lot and just started...He's a very tall, big, muscular guy, and so I had stopped showing up to board meetings in person because I felt very threatened. He was in prison at that time and his girlfriend is the one that filmed me, and then ended up posting it on Facebook.”
**Mental health support**	
3.Therapy/counseling	“Yeah, the healing process is going to take some time. When people went to Vietnam in the war, it took them time. It's crazy that I'm equating this with that, but I'm just like, 'Wow'. We, this small group has been through something not short of a war.”
	“[This] group has been an immense source of support, moral support as well as information and making better decisions. So, it's been invaluable to me.”
	“For whatever reason, my kind of main partner in the pandemic ended up being our county counsel…he is very, very politically astute, not just about the organization, but kind of politics in general…he was really helpful putting things into context again and again…when I would start to feel a little bit like I was just going to lose it, and I just couldn't take one more minute, he would sort of help reground me. Help pull away and think about what we're doing in the context of history…That was really helpful. He just super-steady and very kind…He has done that for me time and time again. So anyway, a cop and a lawyer turned out to be super, super support system.”
4. Vacation/time off work	“I know that there's a commitment for them. They're going to do a retreat, so they're going to have two different weeks so that health officers can try to get away and try to just...You need more than a week. We need sabbaticals. Even if, let's say I were to, hypothetically, leave this job, I need a year off. I don't know. I just need to figure out who I am again.”
	“I think along, as others said, maybe not a full sabbatical, and maybe a month off just would be welcome. Just got to plan it and figure out when that could even happen.”
**Public awareness**	
5. Sharing experiences	“I have talked to the media about it a lot...but the protestors themselves called media attention first. So then it was just, to me, it felt a matter of transparency, but it did help me to talk about it, I think from a therapeutic perspective...After that, I got a huge outpouring of positive feedback, which helped me continue to do my work.”
	“It was structured so that people could articulate their beliefs and be heard. And predicated on an idea that we're not just fundamentally assholes at heart, that we're good people ultimately trying to achieve the same thing, which is healthy, good lives, and be our best. And we love our families, and we love our, all that stuff is still kind of present and American in a lot of ways.
	“And I feel like the media is dividing us. Everything is dividing us…the national conversation needs to be more characterized by a different way of talking about it…And us talking about why we do what we do, where people have an actual chance to how hard it is…from a personal standpoint, rather than being sort of a monolithic mandate-maker. Maybe taking it directly to the national stage and having them hear your stories directly versus, sometimes when it gets filtered through the media takes on a very different slant.”
**Political/Institutional Support**	
6. Support from board of supervisors/state	“It would be helpful to have a civil discourse law on the books in California for government meetings, because our board of supervisor meetings start with 1–3 h every week of rantings, obscenities from people. And then there's just no response and no control. They just let them do it 2–3 min, depending on the day. And it's too much. It shouldn't be allowed to happen that way.”
	“I fell into this not even knowing what the supervisors were, but I just think that the way this plays out in the public arena needs to change. That's just not okay.”
	“So they're sort of always in a bit of a backseat role. And I think that has mostly been helpful, but sometimes it's not helpful, because it means if it's the health officer and a county board of supervisors, like it or not, you have to own a lot of... The sort of public face of the policies and issues.”
7. Support from board of supervisors/state (continued)	“I would say top-level, the board of supervisors has been supportive in that they've not asked me to resign… And they've respected to help out with their role…Their responses are very political because they're politicians. And so if someone's saying something that somewhat echoes some constituents' concerns, then they won't respond. If it's just pure nastiness every now and then, someone will say, once the regular agenda comes back, ‘I'm sorry this is going on. I'm sorry you have to hear this',… But they haven't done anything very full-throated. It's not like they've put out a press release to say, ‘This has got to stop. This is outrageous. We firmly stand behind our public health department and our health officers'. They've not done that. It's been sort of quiet.”
	“I think my board of supervisors thought they knew what their role was and what they should do during a disaster, and turns out I don't think they did... There was opportunity there for the CAO and an OES to step in and really direct them in how to be most effective as an elected official policymaker, rather than trying to run or tell the commanders what to do. I think that's one thing in the future, elected officials, not just our bosses, but city officials for example, an understanding how they can be most effective in a disaster, and allow their appointed directors and officials to do what they're trained to do.”
	“I needed more support from the state, from CDPH and from the governor's office. It feels often like they have no idea what it's really to be like in the field and be boots on the ground. And they seem removed from that and distant…they're so far removed, and they just don't get it. And it felt we were thrown under the bus over and over.”
8. Public support	“When you come into California, being a health officer in California, you come into just this established infrastructure and assumption that health officers relate to each other and that was really great, we leaned hard into that stuff…The implicit support emotionally, but explicitly, just in terms of the defining of the practice, what do we want to do? What are we seeing? All of that.”
	“And then one other note, something happened recently where there was some mask policy changing, actually at the state level. And I was getting a lot of emails, and they were strongly worded in various directions, some pro, some con. And then everything resolved. And somebody, one of those people sent me a thank you, “Thank you for doing nothing”, or something. And it was such a, I had this Stockholm Syndrome moment where it was extremely touching to have somebody, one person, send me a note that was, “Oh, that worked out great for me. Thank you”.”
	“I joined this group ABAJO after the start of the pandemic, but it's hard to imagine what it would've been being in a role like this, that didn't have this type of support network during the pandemic.”
9. Unity among officials	“I drew strength from a number of areas. One is I just think that the people that are working in the public health department in my county are amazing, just amazing….I would get re-inspired when I would see how hard they were working and how creative they were. And I would sort of feel this re-dedication to the mission simply because of the work of colleagues in the department.”
	“And we're all, it's a very supportive environment in that way. And I think it just would've been much harder to be in a smaller department where it's like, and without the external support network where you're sort of on your own, literally. It just doesn't feel like that around here.”
	“And that's, again, why it was so great to have [this group], because we could stick together. I mean, and it was hard to stick together, right? Because we're dealing with, everyone has a different board and a different CAO or CEO and different dynamics.”
10. Increased funding	“At the state level, I think there's more protection because, again, they are insulated in their roles. It's really at the local level that we're going to see this kind of attrition and see this kind of devastation really in the public health programs. Part of it is that they're not funded anyhow. They've never been funded appropriately. We weren't funded before COVID. We have this influx of money. Now we don't have any further funding to support our activities, really, that are significant. I think there's a lot of problems there. I worry, worry very much for local public health.?”
	“Yeah, and I know that we're supposed to get 200 million for local health jurisdictions, ongoing funding once, if the legislature, if they pass it with the budget. That part is great, but whether we're going to be able to hire up, it's not going to be… Just to describe COVID 19-funding, it's like trying to drink from five fire hydrants at the same time, so you're doing all this hiring up. Many, many staff with a lot of fiscal and administrative management.”
	“Then, at the end of the day, you're going to have to pick one-quarter of the people you hire to figure out who gets to stay under this new public health funding. I shouldn't sound like ungrateful for it, but it's just that the reality is that then we're going to downsize again, and the budgets are going to get chipped away.”
	“And that when you don't have infrastructure, and you don't have resources, you don't have a cushion, it's near impossible to adequately respond to a threat like this, like a pandemic or even a fire for that matter. Now, to fix the problem, we need a lot of resources. We need to give public health what it deserves.”

Finally, officials' physical safety must be safeguarded during the completion of their jobs. Many officials were urged to go into their offices unprotected, despite angry riots and protests. A remote option should be available to ensure that officials are not in physical danger.

#### Mental health support

The pandemic sparked several mental health illnesses among public health officials, and there must be support provided for officials to heal as they continue to work. This includes resources allocated toward therapy, counseling, and sabbaticals for officials. Without these interventions, officials' PTSD, depression, and anxiety will only continue to develop and worsen. Participants stated that although this is not a long-term solution to the abuse that they have faced, it would be an opportunity for them to heal after months of stress.

#### Public awareness

Providing public health officials with a space to share their experiences is vital to humanizing this population for the public. It has been a challenge for officials to speak openly about the tough circumstances under which they have worked. Having a non-filtered line of communication between officials and the public would be beneficial for both parties as it could help build an understanding of their frustrations. Officials also shared their positive experiences talking with citizens respectfully and how this has helped to amend their differences in opinions. The media can assist with this process by combating the spread of misinformation.

#### Political/institutional support

Participants reported that the most comprehensive change that could be made was to encourage support throughout several institutions that interface with, support, fund, or regulate local public health departments. In our focus groups, officials frequently discussed the lack of support from their Board of Supervisors. Conflicts between officials and other groups often trickle down to their counties, causing further frustration among citizens.

Furthermore, the officials shared that they were frustrated by politicians who try to appeal to the public rather than to show support. To combat this, the officials stated that leaders must be better educated on public health topics to understand the reasoning behind public health decisions. Law enforcement must also follow in these efforts by ensuring that people respect and follow mandates.

## Discussion

The results from our study are consistent with other literature that illustrates the intense, negative circumstances that public health officials have endured during the pandemic (17, 22, 23). Four major themes were identified to summarize public health officials' experiences during the pandemic, including harassment, psychological impact, systemic backlash, and burnout. Other literature identified similar themes such as under-recognized expertise, disorganized infrastructure, and politicized public health (24). An estimated 59% of officials felt that their expertise was undermined during the pandemic (8, 25), contributing to the isolation that many officials felt in their most recent positions (17, 19, 23).

Officials stated that harassment during the pandemic has been the driving factor of their desire to resign. Since the beginning of the pandemic, an increasing number of American adults believe that harassing public health officials is justified (10, 12). Two-thirds of participants in our study reported feeling harassed in their current or most recent position. In a national survey by the National Association of County and City Health Officials and analyzed by Ward et al., there were 1,499 reports of harassment across 57% of departments, with 43% of officials reporting that they had been individually targeted (11, 18). Another study reports that 41% of public health executives felt bullied, harassed, or threatened by people outside of the health department.

Other studies show that the impact of workplace aggression leads to lower self-efficacy, higher turnover rates, and less job satisfaction (14). Poor mental health has been a leading cause of burnout and depression among public health officials, leading to higher attrition rates (16). In fact, a survey from March 2021 showed that 36.8% of public health workers reported feeling at least one symptom of PTSD in the previous 2 weeks (14). However, 19.6% of public health workers reported needing mental health counseling in the previous month but not receiving it (14).

The CDC reported a similar trend to our results, with the percentage of public health officials who had reported considering leaving their jobs increasing from 44% to 76% before and after the pandemic began, respectively (6). One-third of the public health workforce is estimated to have the intention of resigning in the next year (25, 26). Several participants in our focus groups reported that they had been searching for new positions since the pandemic began and were considering leaving the public health field altogether. These high attrition rates weaken the public health system, especially as new, less experienced individuals fill these roles (19, 23).

Some say that politics is the “public health poison,” as it interrupts how the system works effectively (23). The National Association of County and City Health Officials (NACCHO) found that political pressure had caused 32 agency leaders from 25 departments to resign, change positions, or be fired (18). Officials in our focus groups shared that this pressure had become unbearable, especially when establishing masking or vaccine protocols. Participants shared that their county's political values significantly impacted the extremity of violence that they endured. Funk et al. showed that most Democrats believed that efforts to protect public health have been too scarce (46%), while most Republicans (40%) felt that there was too much priority given to supporting public health (2). Extreme public disapproval has led to national movements to limit health departments' power to make decisions that protect community health (27). As of May 2021, 15 legislatures were considering or had already passed laws to limit the legal jurisdiction of public health agencies (27).

An analysis of our qualitative data showed three main themes for solutions that were discussed: (a) security and protection, (b) mental health support, and (c) political or institutional support. The ongoing stress from fear of personal endangerment is an urgent problem contributing to high rates of burnout in the field (20, 28). To address this, more protective measures must be taken to protect officials and their families.

Many participants felt that there were no punitive actions after they had been harassed or threatened. An estimated 20% of COVID-19 scientists and professionals who reported receiving death threats had employers who were unresponsive or unsupportive (29). To prevent this, there must be strict punishments for those who threaten or harass public health officials (7). Additionally, there must be a better reporting system for acts of violence toward officials. This will help to account for violent incidents and ensure that punitive actions are taken. A 50-statewide survey showed that states vary significantly in their punitive statutes to protect public health officials (30). Creating more cohesive laws that protect public health officials might help to tranquilize national violence toward this targeted population.

Kalmoe et al. showed that when political leaders discourage violence from their followers, it has a strong effect on their behavior (31). Although the politicization of the pandemic has had a negative impact thus far, if more political leaders leveraged their power to stand by public health officials, it could reduce violence in the future (22).

Sharing experiences and communicating openly can also combat the spread of misinformation and increase public awareness of the officials' experiences (1, 23). Evidence shows that when people understand the meaning behind public health policy decisions, there tends to be more support for these policies (26). Therefore, providing public health officials with an opportunity to discuss their decisions may help to ease frustration on a local and state level.

### Limitations

There are several limitations to this study. First, only nine public health officials were included in the focus groups so that participants could feel safe and promote disclosure. Additionally, there was a low number of participants since this was a pilot study, and we plan to recruit more participants in the future. Additionally, it was difficult to obtain more officials for our study due to the anxiety and stress that this population was enduring at the time. This small sample size limits the results of our study as they may not be generalizable to a broader population of public health officials. There is also a geographical limitation as all participants were from counties throughout California. In future research, it would be beneficial to include participants from across the nation. De Beaumont Foundation reported that the public health workforce is 54% white, 18% Hispanic, 15% Black, and 7% Asian (25, 26). However, our sample did not include any Black officials, which may have impacted our results. Despite trying to create a safe environment for participants, there is a possibility that some officials still felt uncomfortable sharing personal experiences from the pandemic. Finally, symptoms of PTSD or other mental illnesses could have resurfaced among participants, making it difficult for them to share their perspectives.

## Conclusion

Our study and other literature show that public health officials have suffered greatly from violence and harassment throughout the pandemic. Our proposed recommendations and solutions provide a multi-faceted approach to the urgent threats that public health officials continue to face. These solutions can be implemented at the local, state, and national levels to spark change throughout the nation. In order to maintain the integrity of the public health system, it is vital that timely changes are made to protect and support public health officials moving forward. Although the COVID-19 pandemic has been a tragedy for many, it offers a unique opportunity to learn from the weaknesses that caused this intense violence toward public health officials. In doing this, we can work with, rather than against, public health officials to strengthen our public health system and improve our response to national emergencies moving forward.

## Data availability statement

This research involved qualitative data that was generated from our various focus groups. We then pulled data from these conversations and included them in our article, including the quotes in Tables 2, 3. However, the raw qualitative data is not able to be shared since it possesses identifiable information from the subjects. Requests to access the datasets should be directed to rita.burke@med.usc.edu.

## Ethics statement

The studies involving human participants were reviewed and approved by University of Southern California, Health Sciences Campus, Institutional Review Board. Written informed consent for participation was not required for this study in accordance with the national legislation and the institutional requirements.

## Author contributions

RB: conceptualized, drafted, and revised the manuscript. AD: drafted, revised, and finalized the manuscript. AA, TM, SD, EH, and LC-K: critically revised the article and drafted the content. All authors contributed to the article and approved the submitted version.
