# Supplementary material for: A qualitative analysis of public health officials' experience in California during COVID-19: priorities and recommendations
Source: Front Public Health. 2023 Sep 13;11:1175661. doi: 10.3389/fpubh.2023.1175661 (PMC10525347; doi:10.3389/fpubh.2023.1175661)
Supplement: Supplementary file 1 [file Data_Sheet_1.pdf]

Appendix:

Focus Group Script:

Dr. Burke: Thank you for agreeing to talk with us today. My name is Dr. Rita Burke and I am here with Anna Distler who will assist me. We have a short survey that we ask that you complete before the start of the focus group.

We would like to learn more about your experience as a public health officer during the COVID-19 pandemic. The focus group will take about 1 hour and will be audio-recorded. You can skip questions that you do not want to answer or stop at any time. We will keep the data we collect confidential and private. We will not share your personal information with anyone outside the research team. Participation is optional. Please tell the facilitator if you do not want to participate.

Study Introduction:

Dr. Burke: The focus group will break down into groups of 8-12 individuals per group. The purpose of this study is to help us understand your experience during the COVID-19 pandemic and how public health officials can be better supported.

Questions:

1. Before we delve into more detailed questions, can you tell me a little about your experience these past 18 months working as a public health official?
2. What has been your biggest challenge?

3. There has been a lot of debate about masks and vaccine mandates. How has that impacted your role?
4. What has had the biggest impact on your ability to perform your day-today-job?
5. It seems that many in public health are leaving their role. Have you considered leaving your role? What made you decide to stay on board?
6. What kind of support did you need during the height of the pandemic? What kind of support would you like to see now?
7. Where have you found support during the pandemic? How have you coped with the backlash against public health? How has this affected your family?

Closing Questions:

1. Is there anything else you would like to share?
2. Do you have any further questions?

Dr. Burke: We appreciate the time you have taken to discuss these questions today. Thank you for your assistance.
